# Autoantibody Biomarkers for Basal Ganglia Encephalitis in Sydenham Chorea and Pediatric Autoimmune Neuropsychiatric Disorder Associated With Streptococcal Infections

**DOI:** 10.3389/fpsyt.2020.00564

**Published:** 2020-06-24

**Authors:** Jennifer L. Chain, Kathy Alvarez, Adita Mascaro-Blanco, Sean Reim, Rebecca Bentley, Rebecca Hommer, Paul Grant, James F. Leckman, Ivana Kawikova, Kyle Williams, Julie A. Stoner, Susan E. Swedo, Madeleine W. Cunningham

**Affiliations:** ^1^Departments of Microbiology and Immunology, University of Oklahoma Health Sciences Center, Oklahoma City, OK, United States; ^2^Section on Behavioral Pediatrics, National Institute of Mental Health (NIMH), Bethesda, MD, United States; ^3^Child Study Center, Yale School of Medicine, New Haven, CT, United States; ^4^Section of Pediatric Neurology, Department of Pediatrics, Yale School of Medicine, New Haven, CT, United States; ^5^Department of Psychiatry, Harvard Medical School, Boston, MA, United States; ^6^Department of Biostatistics and Epidemiology, University of Oklahoma Health Sciences Center, Oklahoma City, OK, United States

**Keywords:** streptococci, autoimmunity, autoantibodies, chorea, obsessive-compulsive disorder, tics, encephalitis, dopamine receptors

## Abstract

Movement, behavioral, and neuropsychiatric disorders in children have been linked to infections and a group of anti-neuronal autoantibodies, implying dopamine receptor-mediated encephalitis within the basal ganglia. The purpose of this study was to determine if anti-neuronal biomarkers, when used as a group, confirmed the acute disease in Sydenham chorea (SC) and pediatric autoimmune neuropsychiatric disorder associated with streptococcal infections (PANDAS). IgG autoantibodies against four neuronal autoantigens (tubulin, lysoganglioside G_M1_, and dopamine receptors D1 and D2) were detected in SC sera (N=8), sera and/or cerebrospinal fluid (CSF) from two groups of PANDAS cases (N=25 first group and N=35 second group), sera from Tourette's syndrome (N=18), obsessive-compulsive disorder (N=25), attention deficit hyperactivity disorder (N=18), and healthy controls (N=28) by direct enzyme-linked immunosorbent assay (ELISA). IgG specific for neuronal autoantigens was significantly elevated during the acute symptomatic phase, and the activity of calcium/calmodulin-dependent protein kinase II (CaMKII) pathway was significantly elevated in human neuronal cells. Five assays confirmed the disease in SC and in two groups of children with PANDAS. In 35 acute onset PANDAS patients, 32 sera (91.4%) were positive for one or more of the anti-neuronal autoantibodies compared with 9 of 28 healthy controls (32.1%, p<0.0001). Importantly, CSF of 32 (91.4%) PANDAS patients had one or more detectable anti-neuronal autoantibody titers and CaMKII activation. Among healthy control subjects with elevated serum autoantibody titers for individual antigens, none (0%) were positively associated with elevated positive CaMKII activation, which was a striking contrast to the sera of PANDAS subjects, who had 76–89% positive association with elevated individual autoantibody titers and positive CaMKII activity. At 6 months follow-up, symptoms improved for more than 80% of PANDAS subjects, and serum autoantibody titers also significantly decreased. Results reported herein and previously published studies in our laboratory suggest the antibody biomarkers may be a useful adjunct to clinical diagnosis of SC, PANDAS, and related disorders and are the first known group of autoantibodies detecting dopamine receptor-mediated encephalitis in children.

## Introduction

Infections and their autoimmune sequelae have been linked to brain pathologies that manifest as adventitious movements and abnormalities of behavior, emotion, and cognition (1–13). Sydenham chorea (SC), the neurological manifestation of acuteo rheumatic fever, is known to be a non-suppurative sequela of group A streptococcal (GAS) infections (14, 15). SC is characterized by the abrupt onset of choreoathetoid movements (16), accompanied by cognitive dysfunction, emotional lability, anxiety, depression, obsessive-compulsive disorder (OCD), and even psychosis (17–19). The psychiatric symptoms appear 2–4 weeks before the onset of chorea, with rates of OCD increasing from 65% to 100% with recurrences of illness (18, 20). These observations suggested that abrupt-onset OCD (in the absence of chorea) might also represent a non-suppurative sequela of GAS infections. A series of clinical studies confirmed this hypothesis, as well as demonstrating emotional, behavioral, and cognitive disturbances similar to those observed in children with SC (21). The unique clinical features of the presentation define the clinical entity, which is known as Pediatric Autoimmune Neuropsychiatric Disorder Associated with Streptococcal infections (PANDAS) (10). PANDAS is characterized not only by the acuity of OCD onset, but also by a complex constellation of co-occurring symptoms, including emotional lability, separation anxiety, adventitious movements [particularly tics and choreiform movements (10, 11)], developmental (behavioral) regression, cognitive decline, and somatic symptoms, including urinary urgency, frequency, and enuresis, as well as insomnia and sleep disruptions. The complex clinical presentation implicated basal ganglia dysfunction in pathogenesis. The close relationship between SC and PANDAS is confirmed not only by the overlapping clinical presentations but also by shared genetic vulnerabilities and a growing body of evidence suggesting that the two clinical presentations share disease mechanisms (10–12, 22).

Both SC and PANDAS are postulated to be caused by an aberrant autoimmune response resulting from molecular mimicry of GAS bacterial and neuronal autoantigens (13, 23–28). The molecular mimicry hypothesis postulates that symptoms arise when antibodies against the dominant streptococcal group A carbohydrate epitope, N-acetyl-beta D-glucosamine (GLcNAc), cross-react with neurons in human basal ganglia (13, 25, 26, 28). Evidence has revealed that these cross-reactive antigens include neuronal surface autoantigens lysoganglioside-G_M1_ and the cytoplasmic α-helical protein tubulin, both of which immunologically mimic GLcNAc (13, 25). Subsequently, autoantibodies derived from SC patients were found to be directed against dopamine D1 and D2L receptors (D1R, D2R, respectively) (26, 29), and were shown to penetrate dopaminergic and other neurons *in vivo* as well as signal the receptor (26). Elevated anti-neuronal autoantibodies were associated with both severity and duration of choreatic episodes, and sera from symptomatic SC patients activated human neuronal cells *in vitro* (13), including signaling of D2R (13, 25, 26, 29). In addition, the ratio of D2R/D1R autoantibody titers in SC correlated with neuropsychiatric symptoms of disease (29). Clinical trials by Garvey et al. and Perlmutter et al. have shown that plasmapheresis and intravenous immunoglobulin (IVIG) decreased chorea severity in SC and improved OCD, tics, and other neuropsychiatric symptoms in PANDAS (30, 31). This collective evidence strongly suggests that both PANDAS and SC are manifestations of basal ganglia encephalitis provoked by cross-reactive anti-neuronal antibodies (26, 29–33).

Animal models provide further support for the clinical role of autoantibodies in SC and PANDAS as passive transfer of anti-streptococcal antibody into mice and rats led to behavioral changes characteristic of both SC and PANDAS (34–37). Expression of the chorea-derived human monoclonal antibody (mAb) 24.3.1 in transgenic mice led to autoantibody targeting of dopaminergic neurons in basal ganglia as well as additional neurons in the cerebral cortex (26). Further, anti-neuronal autoantibodies in sera of PANDAS patients have been shown to target cholinergic interneurons in mouse striatum (38). These interneurons depolarize spontaneously in a manner similar to the cardiac sinoatrial node and help to auto-regulate the local neuronal circuitries (39). The frequency of these spontaneous depolarizations is affected by the activity of dopamine receptors on the surface of cholinergic interneurons in the striatum (39). Thus, development of anti-dopaminergic autoantibodies could dysregulate basal ganglia functions through their impact on cholinergic interneurons. Taken together, evidence from human and animal studies provides strong support for an etiologic role of cross-reactive antibodies in SC and PANDAS and supports the hypothesis that specific antineuronal antibodies might serve as clinically useful biomarkers (40, 41).

The purpose of our study was to evaluate the relationship between a group of anti-neuronal autoantibodies and disease status (acute vs convalescent PANDAS). Serum samples were obtained from two separate cohorts of children with PANDAS [25 patients evaluated at NIMH from 1996 to 1998 (10, 30, 31) and 35 participants of a Yale-NIMH collaborative clinical trial (42)].

## Methods

### Subjects

Samples were obtained from patients and healthy volunteers enrolled in research protocols at NIMH or the Yale Child Study Center. The protocols were reviewed by institutional review boards (IRBs) at the respective institutions: at the NIMH by National Institutes of Health Combined Neuroscience Institutional Review Board, Bethesda, MD, USA; at Yale University, by the Institutional Review Board Human Subjects Committee, New Haven, CT, USA; and at the University of Oklahoma Health Sciences Center by the Institutional Review Board for Protection of Human Subjects, Oklahoma City, OK, USA. In all studies, each parent and child gave written and informed consent or assent, respectively, for the investigation. All parents gave written and informed consent for their children to participate (witnessed by a member of the NIMH human subjects' protection team). All children 7 years and older gave written and informed assent to participate and those 6 and under gave verbal and informed assent. Samples were de-identified and coded to obscure identity and diagnosis prior to shipment.

NIMH provided acute serum samples from eight children with SC (with rheumatic fever) and 25 children with PANDAS evaluated between 1996 and 1998 (10, 30, 31). The SC subjects were identified by independent, direct examinations by two neurologists specializing in movement disorders who identified adventitious choreoathetoid movements that impaired function (30). PANDAS subjects were identified by the following criteria (briefly): presence of OCD and/or a tic disorder, pediatric onset (between 3 years of age and the beginning of puberty), episodic course of symptom severity (abrupt onset of symptoms or dramatic symptom exacerbations, with a decrease in symptom severity between episodes), an association with GAS infection (i.e., associated with positive throat culture and/or elevated anti-GAS antibody titers), and an association with neurological abnormalities [i.e. motoric hyperactivity and adventitious movements, such as tics or choreiform movements (fine piano-playing movements of the fingers)]. Sera were obtained during acute neuropsychiatric symptoms for the PANDAS and SC patients (10). Due to the variable onset of the post-infectious neuropsychiatric sequelae, and delays in referral to the clinical research teams, serum samples were obtained with varying lag-times following the inciting GAS infection. None of the PANDAS or SC subjects had a positive GAS culture at the time sera were collected. NIMH investigators also provided sera from 18 children with chronic symptoms of attention deficit hyperactivity disorder (ADHD) to serve as psychiatric controls. Investigators at the Yale University Child Study Center provided serum samples from 18 children with non-PANDAS Tourette syndrome (TS) and 25 cases of non-PANDAS OCD, all of whom were symptomatic at the time of evaluation. Control samples were provided by 28 healthy subjects evaluated at NIMH, Yale, or Oklahoma simultaneously. To minimize the possibility of false positives, all were free from current infections, pharyngitis, or known psychiatric or autoimmune diseases. Sera were evaluated as soon as possible after collection and were retested repeatedly.

In addition to sera and CSF provided for 25 subjects examined for PANDAS from 1996 to 1998 (first group of 25 samples), serum samples were also provided for 35 participants (12 girls and 23 boys) (second group of 35 samples) of a Yale-NIMH collaborative clinical trial of IVIG for PANDAS (42). All subjects fully met PANDAS diagnostic criteria described above and were moderately-severely ill at baseline. Serum and cerebrospinal fluid (CSF) samples were obtained at baseline and 6 weeks after receipt of IVIG or placebo. Additional serum samples were obtained at 3 and 6 months follow-up. Only the baseline and 6-months samples were analyzed in the investigation shown herein. All assays were conducted in a masked fashion and diagnosis and treatment status revealed only during final data analysis.

### Direct Enzyme-Linked Immunosorbent Assay (ELISA)

Ninety-six-well microtiter plates (Greiner Bio-One, Monroe, NC) were coated with 50 μl of antigen in 100 mM carbonate/bicarbonate buffer (pH 9.6) and stored up to 2 weeks at 4°C. Antigen coating concentrations were as follows: 10 µg/ml of purified porcine tubulin (MP Biomedicals, Santa Ana, CA), 10 µg/ml membrane fragments containing the recombinant human dopamine D1 receptor (D1R, Perkin Elmer, Waltham, MA), 10 µg/ml membrane fragments containing the recombinant human dopamine D2L receptor (D2R, Perkin Elmer), and 20 µg/ml of purified lysoganglioside G_M1_ from bovine brain (Sigma Aldrich, Darmstadt, Germany). Tubulin-, D1R-, and D2R-coated plates were washed three times with phosphate buffered saline (PBS, pH 7.2) containing 0.1% Tween (ThermoFisher Scientific, Waltham, MA). Lysoganglioside-coated plates were washed three times in PBS without Tween in all steps. Plates were blocked with 1% bovine serum albumin (BSA, Roche) in PBS for 60 min at 37°C. Serum or CSF samples serially diluted in 1% BSA (in PBS) were added to washed plates, then incubated overnight at 4°C. The next day, plates were washed as described above and primary IgG antibody binding was detected by adding 50 µl per well of diluted alkaline phosphatase-conjugated goat anti-human γ-chain-specific secondary antibody (polyclonal, Cat# A3312, Sigma Aldrich) and incubated for 60 min at 37°C. The final dilution of secondary antibody was determined empirically for each antigen and validated for every new antibody lot on previously tested samples. Plates were developed at 26°C for 2 h with 50 µl per well of 1 mg/ml p-nitrophenylphosphate (Sigma Aldrich) in 0.1 M diethanolamine buffer (pH 9.8). Optical density values were measured at 405 nm on an automated BioTek microplate reader (BioTek Instruments, Winooski, VT) and corrected by blanks (wells coated with antigen, without serum added). All samples were assayed in duplicate and averaged. Duplicates not matching with ≥20% variance were repeated. Titers represent the serum dilution at optical density of 0.1 at 405 nm after 2 h. Samples with known positive and negative results were included on each plate to standardize and monitor assay performance (26, 29). Each new lot of all reagents and antibodies were validated using serum samples with known titers. Samples were de-identified and coded to obscure identity and diagnosis prior to shipment. Serum samples were periodically re-analyzed by ELISA to maintain standardization of the assays, ensuring control test samples were no more than one titer away from their previous result or the assays were repeated.

### Cell Culture

SK-N-SH human neuroblastoma cells (43) obtained from American Type Culture Collection (ATCC HTB-11, Manassas, VA) were grown in complete F12-Dulbecco's Modified Eagle Medium (ThermoFisher Scientific) as previously described (13). Complete media contained 10% fetal bovine serum (ThermoFisher Scientific) and 1% penicillin-streptomycin antibiotic (ThermoFisher Scientific). Cellular extracts used in the CaMKII assay were centrifuged at 15,000 rpm for 20 min at 4°C. Protein concentrations of the extracts were determined by Bradford assay using the Protein Assay Kit II (Bio-Rad, Hercules, CA) and used to determine specific activity of CaMKII.

### CAMKII Activity Assay

Assay for CaMKII activity was performed as previously described (13). Briefly, SK-N-SH cells were plated in 6-well plates at 2.5 million cells/well and incubated overnight in complete F12-Dulbecco's Modified Eagle Medium, at 37°C with 5% CO_2_. The next day, cells were serum-starved for 30 min in serum-free F12 media with 2 mM CaCl_2_, 2 mM KCl, and 0.4 mM MgCl_2_, then stimulated for 30 min with patient sera or CSF diluted 1:100 in the same media, or with media alone (basal control). Cells were harvested, centrifuged, solubilized in 0.165 ml of protein extraction buffer with protease inhibitors (Soybean Trypsin Inhibitor, Phenylmethanesulfonyl fluoride, Leupeptin, and Aprotinin, Sigma Aldrich, St. Louis, MO), and homogenized. Enzymatic activity was measured using the CaMKII assay system (Promega, Madison, WI) per manufacturer's instructions. Briefly, 5 μl of cell lysate was incubated with 50 μM peptide substrate, buffers and ATP [γ-^32^P] (Perkin Elmer) for 2 min at 30°C. Samples were spotted onto capture membranes and washed. Radioactivity retained on the membrane was measured with a scintillation counter (Beckman Coulter, Indianapolis, IN) and used to calculate specific activity of the CaMKII enzyme (pmol/min/μg) as described in kit instructions. The protein concentration of each sample was used to standardize the CaMKII enzyme activity, and the percentage of specific activity of baseline (basal control) was calculated for each sample where the basal level was set at 100%. All samples were assayed in triplicate and results were averaged. Triplicates not matching with ≥20% variance were repeated. Sera from patients with known high and low CaMKII activity and a basal control sample were included to standardize the assay. Samples were de-identified and coded to obscure identity and diagnosis prior to shipment. Serum samples were periodically re-analyzed for CaMKII activation to maintain standardization of the assays, ensuring control test samples were no more than 20% different from the previous result or the assays were repeated.

Antibody or IgG removal from serum in the CaMKII assay was performed using beads coated with anti-IgG (Sigma Aldrich) or BSA. Beads were diluted to 1 mg/ml in 1% BSA diluent and were mixed with equal volumes of patient sera diluted 1:100 and incubated for 30 min at 37°C, followed by overnight incubation at 4°C with rocking. Sera without beads were diluted 1:200, followed by the same incubation steps. The next day, the sera+beads or sera alone were added directly to plated SK-N-SH cells as described above for the CaMKII assay. All steps for the CaMKII assay were subsequently performed as described above and percent inhibition calculated.

### ASO, ANA, and Anti-DNase B Titers

Antistreptolysin O (ASO), anti-nuclear antibodies (ANA), and anti-DNase B tests were performed by the contributing institutions according to methods previously described using the classical microtiter plate methods with a dilution scheme based on 0.1 log_10_ intervals (44–46).

### Calculation of a Positive Assay and Statistical Analyses

**Figure 1:**
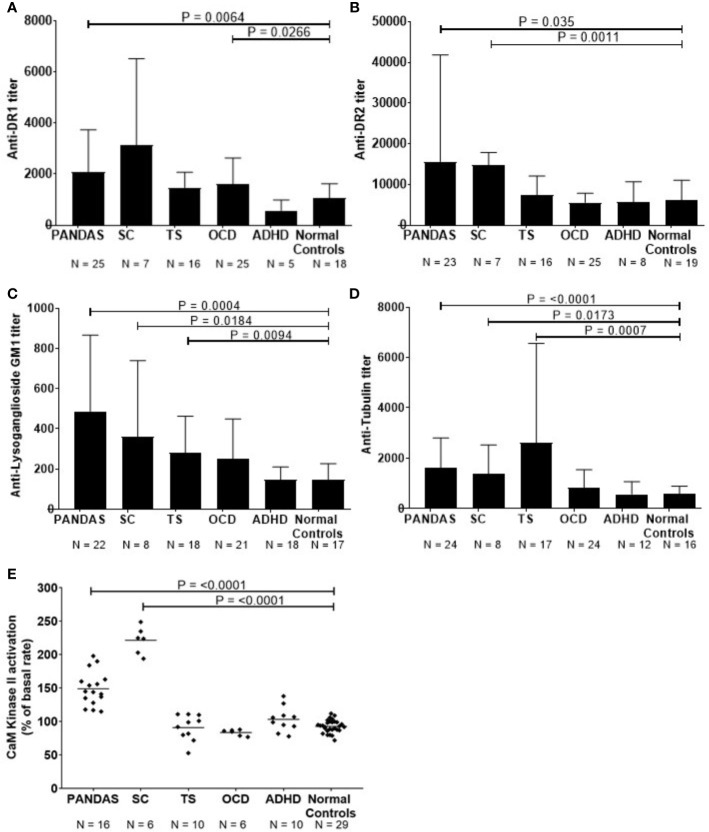
Anti-neuronal autoantibody ELISA titers and CaMKII enzyme activation in childhood neuropsychiatric/movement disorders. **(A)**. Anti-dopamine receptor D1 (D1R) IgG titers, **(B)**. anti-dopamine receptor D2 (D2R) IgG titers, **(C)**. anti-lysoganglioside G_M1_ IgG titers, **(D)**. anti-tubulin IgG titers, and **(E)**. %CaMKII enzyme activation in the human neuronal cell line SK-N-SH above basal level [Figure 1E adapted from (8) with permission from the Journal of Neuroimmunology, Elsevier]. Patients with specific neuropsychiatric/movement disorders include: pediatric autoimmune neuropsychiatric disorder associated with streptococcal infection (PANDAS, from first 50 cases at NIMH^5^), obsessive-compulsive disorder (OCD), and attention deficit hyperactivity disorder (ADHD). Mann-Whitney non-parametric U test performed between each disease group and the normal controls group. An adjusted alpha level of 0.01 was used to account for multiple pair-wise comparisons made between case groups (five different groups) and the normal controls.

A positive serum ELISA titer was established by multiplying the mean of a group of healthy controls times 2 for each of the autoantibodies anti-D1R and anti-tubulin; times 4 for anti-D2R and anti-lysoganglioside G_M1_. These calculations were determined based on checkerboard titrations to accurately separate normal from disease reactivity. Based on these calculations, a positive serum titer result for anti-D1R was set at ≥ 4,000, for anti-D2R ≥ 16,000, for lysoganglioside G_M1_, ≥ 640, and for tubulin ≥ 2,000. A positive serum result for the antibody-mediated CaMKII activation was ≥ 130% or three standard deviations above basal mean activation rate. Also, the normal range was based on results as shown in **Table 3** (72–112%) and **Figure 1E** where the normal range approached 125% above basal rate. For CSF, a positive CaMKII activation threshold was established as 100% using normal CSF activation range (70–80%) (13). CSF positive titer results were ≥ 5 for all antigens in the ELISA. Positive results for ASO were determined as ≥ 167 Todd units (44–46). Positive anti-DNase B results were > 375. Positive ANA results were determined by the Mayo Clinic standard of > 1. Statistical significance is defined as a P value ≤ 0.05, as determined by Mann-Whitney (non-parametric) *U* test for comparison of the median between independent groups and by the Wilcoxon signed-rank test for comparison between paired groups. Non-parametric testing approaches were used because normality of the distribution of means could not be assumed under the Central Limit Theorem given the non-normal distributions of the antibody titer measures and the available sample sizes (47). An adjusted alpha level of 0.01 was used to adjust for multiple pair-wise comparisons made between case groups (five different groups) and the normal control groups based on a Bonferroni correction for the five pairwise comparisons of interest specified *a priori*. Proportions were compared between independent groups using a Fisher's exact test when expected frequencies were less than 5 for more than 20% of the cross-tabulated categories (47).

## Results

### Anti-Neuronal Autoantibodies in SC and PANDAS

Using direct ELISA, we evaluated reactivity of sera from children with SC (n = 8) or PANDAS (first group) (n = 25) against four autoantigens dopamine receptors D1R and D2R (26, 29), lysoganglioside G_M1_ (8, 13), and tubulin (25) tested in the direct ELISA. **Figure 1** shows serum results for the patient groups compared to healthy volunteers. As shown, acute-onset PANDAS sera had significantly elevated IgG autoantibody titers against dopamine receptors D1R (P = 0.0064, **Figure 1A**) and D2R (P = 0.035, **Figure 1B**), lysoganglioside G_M1_ (P = 0.0004, **Figure 1C**), and tubulin (P = <0.0001, **Figure 1D**). Consistent with previous studies (8, 13, 25, 29), sera from patients with SC demonstrated significantly elevated anti-neuronal autoantibodies against D2R (P = 0.0011, **Figure 1B**), lysoganglioside G_M1_ (P = 0.0184, **Figure 1C**), and tubulin (P=0.0173, **Figure 1D**) compared to healthy controls. ADHD subjects did not have elevated autoantibodies, OCD subjects had elevated autoantibodies against D1R antigen (P=0.0266, **Figure 1A**) and TS subjects had elevated autoantibodies against lysoganglioside G_M1_ (P=0.00094, **Figure 1C**) and tubulin (P=0.0007, **Figure 1D**), which supports previous studies demonstrating inflammation in the basal ganglia in TS (48, 49). In **Figure 1**, compared to healthy controls, significant differences in IgG autoantibody titers are distinct for the four individual neuronal antigens tested for each of the five patient groups. Although the limited sample size is insufficient for a rigorous breakdown of phenotypes, there were general trends of more positive D2R autoantibodies in SC (6 out of 7) and more positive D1R autoantibodies in PANDAS. The data further suggested potential elevation of tubulin autoantibodies in tics and D1R in OCD. These trends where certain antigens were more positive in certain pathologies is interesting and potentially important in our understanding of basal ganglia encephalitis but the small sample size does limit the power of the study. The four neuronal autoantibody specificities represent a group of autoantibodies present in basal ganglia encephalitis with patterns of overlap of the autoantibodies in different pathologies (SC, PANDAS, TS, and OCD) that affect the basal ganglia. PANDAS patients have these reflected in the four antigens recognized in the autoantibody group due to their potentially multiple neuropsychiatric pathologies. For the CaMKII results in **Figure 1**, TS, OCD, and ADHD controls were negative. At the time that these TS, OCD, and ADHD samples were collected when the first 50 cases were enrolled at NIMH, the TS, and OCD controls were not acute onset or related to GAS infections and autoimmunity. Further studies are needed to sort out the complexities of Tics, OCD, and ADHD in the clinical setting where an acute onset or infection is not known or present.

In addition to the direct ELISA, serum antibody activation of the CaMKII enzyme in the human neuronal cell line SK-N-SH provides a significant advantage by detecting functionally signaling autoantibodies in children with disease compared to healthy control subjects (13). When CaMKII antibody-mediated activation was studied in patients with neuropsychiatric disorders such as PANDAS, TS, OCD, SC, and ADHD compared to healthy subjects, it was significantly elevated in PANDAS and acute SC (**Figure 1E**, P = <0.0001). Children diagnosed with PANDAS demonstrated antibody-mediated CaMKII enzyme activation at a mean of 150% (range = 115–198%) and those with SC had CaMKII activation of 221% (range = 194–249%) compared to normal control subjects with a mean CaMKII activation of 93% (range = 72–112%). Sera from the children diagnosed with non-PANDAS TS, OCD, or ADHD demonstrated normal levels of CaMKII activation (**Figure 1E**), despite the presence of elevated autoantibodies in some of their sera.

### Results From Second Group of PANDAS Sera (35 Cases)

Sera from 35 children acutely ill with PANDAS were evaluated for reactivity to the group of four neuronal autoantigens (D1R, D2R, lysoganglioside G_M1_, and tubulin) in the direct ELISA and in the human neuronal cell antibody-mediated CaMKII activation assay. The individual test results for each patient are shown in **Table 1**. At baseline, 71.4% of the sera were positive (≥ 4,000) for antibodies against dopamine receptor D1R, 25.7% were positive (≥ 16,000) for antibodies against dopamine receptor D2R, 17.1% were positive (≥ 640) for antibodies against lysoganglioside G_M1_, 28.6% were positive (≥ 2,000) for antibodies against tubulin, and 71.4% were positive (≥ 130%) for CaMKII activation in the human neuronal cell line. Together, the group of assays was sensitive enough to detect at least one positive test result in 32 of 35 PANDAS patients, or 91.4%. Other antibody tests also were performed, including ASO and anti-streptococcal DNAse B (anti-DNaseB) which are used clinically to detect recent streptococcal infection, and antinuclear antibody (ANA) which is used for autoimmune disorders (44–46, 50, 51). Positive ASO titers were found in 60.0% of the serum samples, 37.1% were positive for anti-DNAseB, and 13 sera (37.1%) had positive ANA titers (**Table 1**). Among the 10 patients who had negative results for all three clinical assays, nine had at least one positive test in the autoantibody and CaMKII activation assays. Thus, the group of anti-neuronal autoantibody assays identified 91.4% of patients with PANDAS symptoms compared to 60.0%, 37.1%, and 37.1% respectively for ASO, anti-DNase B assays, or ANA.

**Table 1:** Autoantibody ELISA titers and antibody-mediated CaMKII activation results in sera from patients diagnosed with PANDAS.

Patient #	α-D1R	α-D2R	α-Lysoganglioside-G_M1_	α-Tubulin	CaMKII Activation	ASO	ANA	Anti-DNase B
**1**	**16,000**	**16,000**	160	1,000	**154.0**	0	0	0
**2**	**8,000**	4,000	160	1,000	112.0	**403**	1.0	**397**
**3**	**16,000**	**16,000**	320	**2,000**	**149.0**	**180**	0	103
**4**	2,000	4,000	**640**	**2,000**	**136.7**	**692**	**1.7**	**650**
**5**	2,000	4,000	160	1,000	**177.7**	**267**	0	**580**
**6**	2,000	2,000	160	1,000	**145.7**	0	**1.6**	0
**7**	**4,000**	4,000	160	1,000	120.8	**206**	**1.1**	126
**8**	**4,000**	8,000	160	**2,000**	**192.3**	**575**	**1.4**	**956**
**9**	1,000	2,000	160	1,000	**168.5**	36	0	112
**10**	**8,000**	**16,000**	320	**2,000**	**216.5**	0	**2.0**	106
11	1,000	2,000	320	500	108.4	39	0	194
**12**	**4,000**	4,000	20	500	109.0	0	0	96
**13**	**4,000**	4,000	160	500	**136.0**	**283**	0	**450**
**14**	**4,000**	4,000	160	1,000	127.0	0	0	0
**15**	**8,000**	**16,000**	160	500	**173.0**	0	**2.0**	0
**16**	**8,000**	**16,000**	320	500	**169.0**	**485**	0	205
**17**	**8,000**	8,000	**640**	**2,000**	**125.0**	**379**	0	104
**18**	**16,000**	**16,000**	320	**2,000**	**167.0**	**843**	**1.8**	**420**
**19**	**4,000**	4,000	320	**2,000**	112.0	**601**	0	**615**
**20**	**4,000**	4,000	320	1,000	**197.0**	37	0	196
**21**	2,000	**16,000**	320	**2,000**	**152.3**	**455**	0	366
**22**	2,000	8,000	320	1,000	**204.5**	**395**	**1.2**	164
**23**	**8,000**	8,000	**640**	1,000	**178.6**	**533**	**1.2**	0
**24**	**4,000**	8,000	**640**	1,000	**179.6**	**307**	0	**499**
**25**	**4,000**	**16,000**	**640**	**2,000**	95.1	96	**4.3**	**642**
**26**	2,000	2,000	160	**2,000**	**160.2**	20	0	0
**27**	**4,000**	4,000	160	500	**180.1**	55	0	352
**28**	**4,000**	8,000	20	1,000	**187.6**	**671**	**1.3**	**629**
**29**	**4,000**	8,000	160	1,000	**189.6**	**183**	**1.6**	286
**30**	**4,000**	8,000	**640**	1,000	**209.4**	**265**	0	**617**
31	1,000	2,000	80	500	124.4	0	0	0
**32**	**4,000**	4,000	160	500	**144.0**	**174**	0	185
**33**	**4,000**	4,000	160	1,000	**133.0**	0	0	88
**34**	**8,000**	**16,000**	160	500	**157.9**	**1110**	**1.2**	**1310**
35	1,000	8,000	320	1,000	128.6	**875**	0	**624**
**Number of positive results**	**25/35**	**9/35**	**6/35**	**10/35**	**25/35**	**21/35**	**13/35**	**13/35**
**Percent of positive results**	**71.4%**	**25.7%**	**17.1%**	**28.6%**	**71.4%**	**60.0%**	**37.1%**	**37.1%**
**Total panel positivity**	**32/35 PANDAS cases were positive (91.4% sensitivity in SERA)**							

D1R, dopamine receptor D1, positive ≥ 4,000; D2R, dopamine receptor D2L, positive ≥ 16,000; lysoganglioside, positive ≥ 640; tubulin, positive ≥ 2,000; CaMKII, calcium/calmodulin-dependent protein kinase, positive ≥ 130%; ASO, anti-streptolysin O, positive ≥ 167; ANA anti-nuclear antibodies, positive = >1; anti-DNase B, positive >375.

**Bold** = positive result or neuronal panel-positive patient.

“0” = undetectable level of antibody.

The PANDAS subjects underwent lumbar puncture at baseline and CSF samples were tested for the presence of anti-neuronal autoantibody biomarkers and antibody-mediated CaMKII activity. As shown in **Table 2**, autoantibodies against D1R were detectable in 10/34 CSF samples (29.4%, CSF titer range = 5-250); 22/34 (64.7%) had antibodies against D2R (CSF titer range = 5–80); 2/27 (7.4%) against lysoganglioside G_M1_ (CSF titer range = 5–10), and 4/32 (12.5%) against tubulin (CSF titer range = 5–20). CaMKII activation was detectable in the CSF of 24/34 subjects (70.6%; range = 104–171% above basal activity). In total, 32 of 35 PANDAS CSF samples (91.4%) had at least one positive test; 13 had one positive assay, 11 had two, 6 had three, 1 had four, and 1 sample was positive for all five assays. Results demonstrate anti-neuronal autoantibodies which can activate neuronal cells are detectable in CSF diluted 1:100. We diluted CSF 1:100 and retained positive activity (≥100–171% of basal activity) which was not as strong as observed in sera where a positive sample was ≥130–>200% of basal activity. Although we did not assay normal CSF in this study, we have found in our previous studies where normal CSF was available that normal CSF signaling in the CaMKII assay was ≤ 60% of basal activation of human neuronal cells (13). In **Table 2**, CSF CaMKII activation remained above 100% in 91.4% of the PANDAS cases.

**Table 2:** Autoantibody ELISA titers and antibody-mediated CaMKII activation results in CSF* from patients diagnosed with PANDAS.

Patient #	α-D1R	α-D2R	α-Lysoganglioside-G_M1_	α-Tubulin	CaMKII Activation	At least one positive result?	
						Serum	CSF
**1**	20*	10*	<5	ND	95	Yes	Yes
**2**	<5	**5**	<5	<5	**121**	Yes	Yes
**3**	10*	20*	<5	<5	69	Yes	Yes
**4**	<5	ND	ND	<5	154*	Yes	Yes
**5**	<5	<5	<5	<5	144*	Yes	Yes
**6**	<5	<5	<5	<5	141*	Yes	Yes
**7**	<5	<5	<5	<5	**104**	Yes	Yes
**8**	ND	<5	<5	ND	110*	Yes	Yes
**9**	<5	**5**	<5	**5**	99	Yes	Yes
**10**	20*	80*	**10**	20*	122*	Yes	Yes
**11**	<5	**5**	<5	**5**	**121**	No	Yes
**12**	<5	<5	ND	<5	**121**	Yes	Yes
**13**	<5	<5	<5	<5	114*	Yes	Yes
**14**	<5	**5**	<5	<5	**104**	Yes	Yes
**15**	5*	20*	<5	<5	123*	Yes	Yes
**16**	5*	**5**	ND	<5	115*	Yes	Yes
**17**	<5	**20**	<5	<5	110*	Yes	Yes
**18**	10*	40*	**5**	<5	69	Yes	Yes
**19**	<5	**5**	<5	<5	ND	Yes	Yes
**20**	5*	**5**	ND	<5	124*	Yes	Yes
**21**	<5	5*	<5	<5	136*	Yes	Yes
**22**	<5	**5**	<5	<5	96	Yes	Yes
**23**	5*	**5**	<5	<5	171*	Yes	Yes
**24**	<5	<5	<5	<5	113*	Yes	Yes
**25**	<5	5*	<5	<5	**127**	Yes	Yes
**26**	**5**	**5**	<5	5*	145*	Yes	Yes
**27**	<5	**5**	<5	<5	98	Yes	Yes
**28**	<5	**5**	<5	<5	107*	Yes	Yes
**29**	<5	**5**	ND	ND	147*	Yes	Yes
**30**	<5	**5**	<5	<5	142*	Yes	Yes
31	<5	<5	<5	<5	99	No	No
**32**	<5	<5	ND	<5	121*	Yes	Yes
**33**	250*	<5	ND	<5	92	Yes	Yes
34	<5	<5	ND	<5	96	Yes	No
35	<5	<5	<5	<5	88	No	No
**Number of Positive Results**	**10/34**	**22/34**	**2/27**	**4/32**	**24/34**	**33/35 =**	
**Percent of Positive Results**	**29.4%**	**64.7%**	**7.4%**	**12.5%**	**70.6%**	
**Total panel positivity**	**32/35 PANDAS cases were positive (91.4% sensitivity in CSF)**					**94.3%** **Match with Sera**	

Diluted 1:100.

ND, not determined (limited sample); <5, below detectible limits; D1R, dopamine receptor D1, positive ≥ 5; D2R, dopamine receptor D2L, positive ≥ 5; lysoganglioside, positive ≥ 5; tubulin, positive ≥ 5; CaMKII, calcium/calmodulin-dependent protein kinase, positive ≥ 100%.

**Bold** = positive result, positive patient.

Yes = One or more positive result.

No = No positive results.

* = Positive result also seen in sera from same patient.

When we directly compared results from serum and CSF samples (diluted 1:100), we found that 33 out of 35 (94.3%) subjects had matched results, with 31 subjects displaying one or more positive results in both serum and CSF (**Table 2**, right columns). For example, subject #10 displayed high antibody titers for all neuronal autoantigens and CaMKII activation in both serum and CSF, with others also showing multiple positive results in both serum and CSF. Only two of 35 subjects (5.7%) had serum-CSF results which did not match. One subject (#11) only had detectable autoantibodies in CSF and another subject (#34) only had detectable autoantibodies in serum.

Although individual PANDAS patients of the second group had matching sera and CSF based on at least one positive result in the five assays, not every positive neuronal antigen in the serum ELISA matched with the CSF ELISA when the four autoantigens were considered individually. See **Table 2** (*) for where the CSF and sera positivity for that antigen matched exactly. The percent positivity for each of the four antigens tested is shown at the bottom of **Table 2**. There were 12 CSF that did not have a positive ELISA with any of the four autoantigens while 10 of those 12 CSF did have a positive result in the neuronal cell activation assay, the CaMKII which provided nearly 100 percent correlation of the serum and CSF reactivities with the five assays. Because there were clearly more positives in the D1R and D2R autoantibody groups, it was interesting to note that the D2R antibody positivity was strikingly higher (64.7%) in the CSF compared to the sera (25.7%) and could suggest that the D2R autoantibody concentrated in the CSF compared to the serum either by IgG or lymphocyte/plasma cell leakage across the blood brain barrier (BBB). Although there was a clear D1R autoantibody preference in serum vs D2R autoantibody preference in CSF, it is difficult to know the effects of D1R vs D2R and the contribution of their ratio and avidity as well as the receptors as displayed in individuals with disease. The ratio of these two autoantibodies has already been shown to correlate with symptoms in a previous study of SC (30). Further the positivity of anti-lysoganglioside and anti-tubulin antibodies could be additive affect the outcome in the disease, but this is not yet well established.

In summary for **Tables 1** and **2**, our data demonstrate the presence of elevated anti-neuronal autoantibodies in both the serum and CSF of 94.3% of PANDAS patients, supporting the hypothesis that autoantibodies (IgG) or lymphocytes producing these autoantibodies can cross the BBB, bind to neuronal antigens, and signal neuronal cells. The importance of positive CSF in PANDAS supports an inflammatory pathogenesis in the brain which may be described as an encephalitis (52), and the study of magnetic resonance imaging (MRI) in PANDAS has previously demonstrated inflammation in the basal ganglia (40).

### Results From Healthy Volunteers

Sera from 28 healthy subjects were also tested for autoantibodies against the neuronal autoantigens in the direct ELISA, as well as in the human neuronal cell CaMKII activation assay. In healthy controls, the mean titer calculated for anti-D1R was 1,096, for anti-D2R 6,000, for anti-lysoganglioside 147, and for anti-tubulin 956. Individual test specificities showed that 85.7% of healthy subjects were negative (≤2,000) for antibodies against dopamine receptor D1R, 85.7% were negative (≤8,000) for antibodies against dopamine receptor D2R, 96.4% were negative (≤320) for antibodies against lysoganglioside G_M1_, 96.4% were negative (≤1,000) for antibodies against tubulin, and 100% were negative (≤129%) for CaMKII activation (**Table 3**). Nineteen out of 28 healthy subjects (67.8%) had completely negative results for the group of anti-neuronal autoantibody assays, in other words, 32% of healthy subjects had at least one elevated autoantibody ELISA test in the group. The CaMKII was negative in all healthy control subjects in the study.

**Table 3:** Autoantibody ELISA titers and antibody-mediated CaMKII activation results in healthy subjects.

Patient #	α-D1R	α-D2R	α-Lysoganglioside-G_M1_	α-Tubulin	CaMKII Activation	ASO
**1**	500	**16,000**	160	1,000	93	87
**2**	1,000	**16,000**	160	1,000	100	**221**
3	2,000	4,000	80	500	99	166
**4**	1,000	**16,000**	80	500	98	79
5	500	2,000	200	500	89	**513**
6	500	2,000	100	250	94	**271**
7	1,000	2,000	200	250	98	**487**
8	500	2,000	100	500	92	70
9	2,000	4,000	200	1,000	106	**421**
10	1,000	8,000	100	1,000	99	78
11	1,000	2,000	80	500	88	**240**
12	1,000	4,000	80	500	96	**200**
13	1,000	4,000	80	500	94	**200**
14	500	2,000	160	250	86	**200**
15	2,000	8,000	80	500	99	**320**
16	1,000	4,000	80	1,000	53	25
**17**	2,000	8,000	320	**2,000**	95	35
18	1,000	2,000	80	500	79	63
19	1,000	4,000	80	500	87	**200**
20	2,000	8,000	80	1,000	80	25
**21**	**4,000**	8,000	**1280**	1,000	72	25
**22**	**4,000**	8,000	320	1,000	88	25
**23**	**8,000**	4,000	320	1,000	90	25
**24**	2,000	**16,000**	320	500	112	**250**
**25**	**8,000**	4,000	160	500	80	25
26	2,000	4,000	160	500	100	50
27	2,000	2,000	160	1,000	92	160
28	1,000	1,000	80	500	104	125
**Number of Negative Results**	**24/28**	**24/28**	**27/28**	**27/28**	**28/28**	**16/28**
**Percent of Negative Results**	**85.7%**	**85.7%**	**96.4%**	**96.4%**	**100%**	**57.1%**
**Total Panel Specificity**	**19/28 healthy subjects were negative (67.8% specificity)**					

D1R, dopamine receptor D1, positive ≥ 4,000; D2R, dopamine receptor D2L, positive ≥ 16,000; Lyso, lysoganglioside GM1, positive ≥ 640; tubulin, positive ≥ 2,000; CaMKII, calcium/calmodulin-dependent protein kinase, positive ≥ 130%; ASO, anti-streptolysin O, positive ≥ 167.

Bold = positive result or neuronal panel-positive subject.

**Figure 2:**
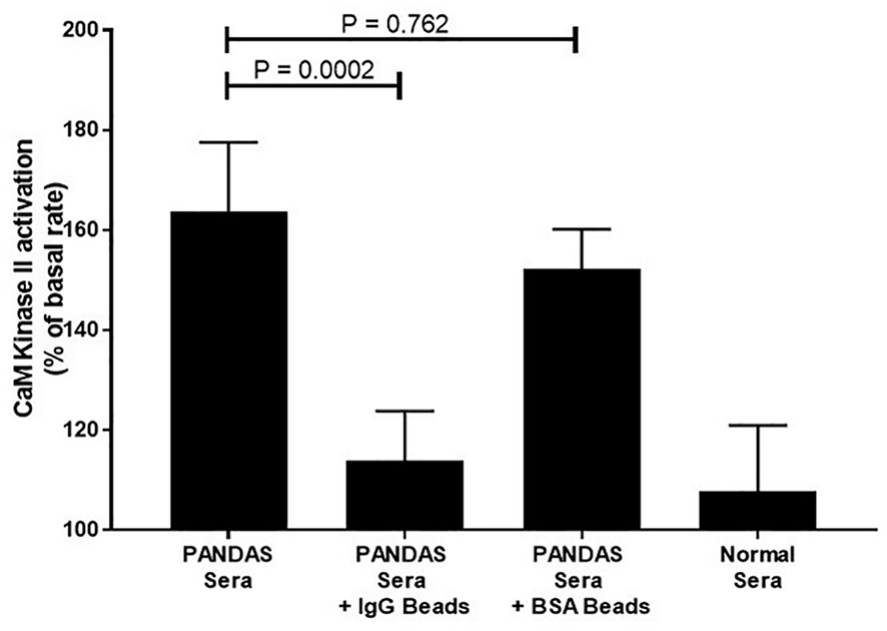
Anti-IgG inhibited PANDAS serum activation of CaMKII in a human neuronal cell line. Diluted sera (1:100) from two subjects diagnosed with PANDAS were incubated in triplicate with no beads, anti-IgG-coated beads, or BSA-coated beads and analyzed for CaMKII activation. Sera from one normal subject was also analyzed for CaMKII activation. Mann-Whitney non-parametric U test performed. ns, not significant.

**Table 4** summarizes the positive autoantibodies in the 35 PANDAS subjects compared to the 28 healthy controls. The comparison showed statistically elevated autoantibodies against D1R (P<0.0001) and tubulin (P=0.0094), as well as elevated autoantibody-mediated CaMKII activation (P<0.0001) in the entire PANDAS cohort. Treatment of selected PANDAS sera with anti-IgG beads removed IgG antibodies in the sera and thus reduced the CaMKII activation to that of normal sera (**Figure 2**).

**Table 4:** Comparison of percent positive anti-neuronal autoantibody ELISA assays, antibody-mediated CaMKII activation, and anti-streptolysin O assay results in PANDAS vs healthy subjects.

**Autoantigen**	**PANDAS** **(N = 35)**		**Healthy Subjects** **(N = 28)**		**P-Value**
	**#** **Positive**	**%** **Positive**	**#** **Positive**	**%** **Positive**	
Anti-D1R	25	71.4%	4	14.3%	<0.0001
Anti-D2R	9	25.1%	4	14.3%	0.27
Anti-lysoganglioside-G_M1_	6	17.1%	1	3.6%	0.12*
Anti-tubulin	10	28.6%	1	3.6%	0.0094
CaMKII activation	25	71.4%	0	0.0%	<0.0001*
ASO	21	60.0%	12	42.9%	0.18

*Fisher's exact test.

PANDAS, pediatric autoimmune neuropsychiatric disorder associated with streptococcal infections; D1R, dopamine receptor D1; D2R, dopamine receptor D2L; CaMKII, calcium/calmodulin-dependent protein kinase; ASO, anti-streptolysin O.

When children with PANDAS had elevated serum autoantibodies against each of the individual four neuronal autoantigens, they most often had positive CaMKII functional activity, while healthy control subjects with elevated serum autoantibodies did not (summarized in **Table 5**). Data from **Table 1** showed that among the 25 subjects with PANDAS who had elevated D1R titers (≥4,000), 19 also had positive (≥130) CaMKII activation (76%). Eight of nine PANDAS subjects with elevated D2R titers (≥16,000) had positive CaMKII activation (89%), as did five of six with elevated lysoganglioside-G_M1_ titers (≥320, 83%) and 8 of 10 with elevated tubulin titers (≥2,000, 80%). The same trend was observed between individual autoantibody titers and CaMKII in CSF (**Table 2**), although not quite as high as in sera of the same subjects (60% for D1R, 68% for D2R, 50% for lysoganglioside-G_M1_, and 75% for tubulin, as summarized in **Table 5**). Most importantly, among healthy control subjects who had elevated serum autoantibody ELISA titers for individual antigens, none (0%) were positive for CaMKII activation, which is a striking contrast to the sera of PANDAS subjects, who had 76–89% elevated individual autoantibody ELISA titers and positive CaMKII activity (**Tables 1** and **3**, summarized in **Table 5**). Clearly, elevated individual ELISA titers were concomitantly elevated with positive functional CaMKII activity in disease subjects but not in healthy controls.

**Table 5:** Elevated anti-neuronal autoantibody ELISA titers were associated with positive CaMKII activation in disease subjects but not in healthy controls.

**Autoantigen**	**PANDAS** **Sera** **Dual Positive** **w/CaMKII** (**Table 1**)		**PANDAS** **CSF** **Dual Positive** **w/CaMKII** (**Table 2**)		**Healthy** **Subjects** **Dual Positive** **w/CaMKII** (**Table 3**)	
	**#**	%*	**#**	%*	**#**	%*
Anti-D1R	19/25	76%	6/10	60%	0/4	0%
Anti-D2R	8/9	89%	15/22	68%	0/4	0%
Anti-lysoganglioside-G_M1_	5/6	83%	1/2	50%	0/1	0%
Anti-tubulin	8/10	80%	3/4	75%	0/1	0%

*Percent CaMKII positive

PANDAS, pediatric autoimmune neuropsychiatric disorder associated with streptococcal infections; D1R, dopamine receptor D1; D2R, dopamine receptor D2L; CaMKII, calcium/calmodulin-dependent protein kinase, positive ≥130 for sera, ≥100 for CSF.

### Six Months Follow-Up of Second Group (n=35) of PANDAS Cases Reveals Symptomatic Improvement Associated With Reduced Anti-Neuronal Autoantibody ELISA Titers and CaMKII Activation.

**Figure 3:**
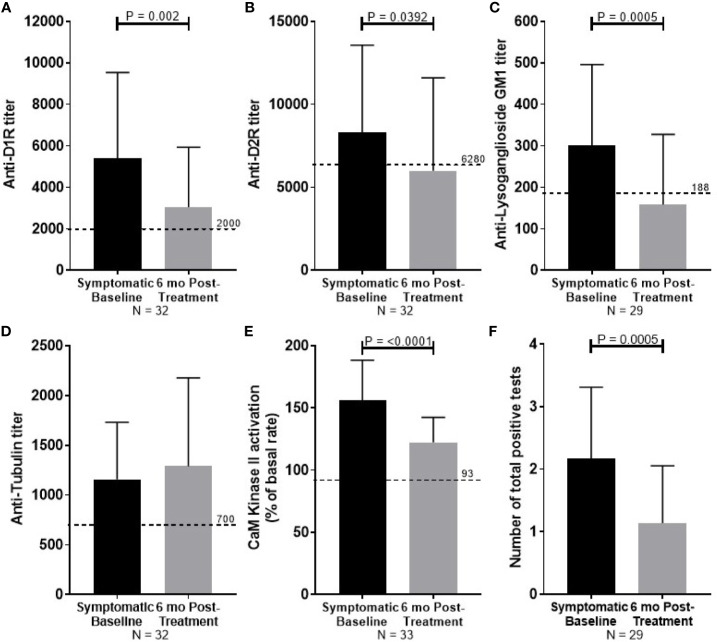
Summary of serum autoantibody ELISA titers and antibody-mediated CaMKII activation results of PANDAS study subjects at diagnosis (black bar) and after improvement (gray bar). Sera were examined from subjects diagnosed with PANDAS at the NIMH at the time of diagnosis (symptomatic baseline) and at symptom improvement at 6 months (6 month post-treatment). **(A)** Anti-dopamine receptor D1 (D1R), **(B)** anti-dopamine receptor D2 (D2R), **(C)** anti-lysoganglioside G_M1_, **(D)** anti-rubulin, **(E)** calcium/calmodulin-dependent protein kinase II (CaMKII) activation. **(F)** The number of total positive tests per patient before and after treatment are shown. Dotted line represents the mean titer result for normal controls. Wilcoxon signed-ranked test performed.

At 6 months follow-up, symptom severity was decreased for all 35 PANDAS patients, with 80% reported to be “much improved” or “very much improved”. Significant reduction in autoantibody titers was observed for anti-D1R (P = 0.002, **Figure 3A**), anti-D2R (P = 0.0392, **Figure 3B**), and anti-lysoganglioside G_M1_ (P = 0.0005, **Figure 3C**). Reduction in antibody-mediated CaMKII activation (P <0.0001, **Figure 3E**) was also observed with improvement. Tubulin titers were not significantly reduced (**Figure 3D**). The number of positive tests for each convalescent serum sample was reduced from the number of positive tests in the child's acute sera (P = 0.0005, **Figure 1F**).

## Discussion

Historically, the etiologic role of GAS infections in SC has long been recognized (14, 15, 19, 28, 29). More recently, GAS infections have been linked to the abnormal movements and behaviors of PANDAS (8, 10–12, 53–55). In both disorders, evidence suggests that neuroinflammation may result from a process known as molecular mimicry, where epitopes are shared between host and pathogen (28). Human monoclonal antibodies in sera and CSF derived from SC demonstrated cross-reactivity between streptococcal and neuronal antigens (13, 25, 26), and antibodies in PANDAS sera and CSF cross-reacted with both microbial and neuronal antigens (8). The ability of cross-reactive autoantibodies to access the CSF and brain is a critical effector component to the pathogenesis of autoimmune encephalitis. There is increasing evidence that the components of the peripheral immune system are able to cross the BBB and enter the central nervous system (CNS) (52, 56–60) when permeability of the BBB is compromised by infectious or non-infectious factors (57, 58). Bacteria, including group A streptococci, can impair BBB function in neurological niches like the olfactory bulb leading to loss of function, i.e. odor processing (52). In these recent studies, Th17 lymphocytes promoted vascular and neurological deficits in a mouse model of GAS infection induced encephalitis. In this model, multiple GAS infections led to Th17 lymphocyte extravasation from the nose into the brain causing BBB breakdown with IgG entering the CNS and loss of excitatory synapses within the olfactory bulb (58). Th17 cells in the upper respiratory tract mucosa are activated by extracellular pathogens, such as group A streptococci, may promote autoimmune sequelae with autoantibodies and are closely associated with activated neutrophils which lead to clearance of extracellular bacterial pathogens. Further, inflammatory cytokines and chemokines may also impair the stability of the BBB by disrupting tight junction proteins. The upregulation of certain adhesion molecules such as VCAM-1, ICAM-1, and ICAM-2 on CNS vessels during inflammation can promote the trafficking of lymphocytes into the CNS. Antibody-secreting B cells are found in the brain and CSF in infectious neurological diseases as well as in demyelinating diseases (59). One study suggested short-term sleep deprivation led to an influx of B cells across the BBB through a mechanism involving CXCR5 (60), a chemokine linked to BBB permeability (61). Inflammation in basal ganglia was demonstrated in PANDAS cases by PET imaging (40) and by mRNA analysis of post-mortem specimen of affected individuals (62). Our results are consistent with these mechanisms proposed, and the actual *in vivo* pathology may be multifactorial, involving several immune processes that occur during inflammation, such as BBB permeability due to cytokine signaling, trafficking of autoantibody-producing B cells and T cells to the CNS, promoting microglial damage to tissue, in addition to autoantibody-mediated signaling with alterations in movement and behavior.

Although anti-neuronal autoantibody titers were lower in the CSF than in the sera, their presence in CSF of PANDAS subjects is evidence of neuronal inflammation during symptomatic episodes and demonstrates the ability of these auto-antibodies to cross the BBB and potentially bind to neuronal antigens. Antibodies of any kind are not typically detected in CSF of healthy individuals. In cases where antibodies are detected in CSF but not serum, the differences may be related to delayed clearing of antibodies from the central nervous system (63). It could also be related to the timing of CSF vs serum collection, the presence of B cells secreting antibody in the CSF, the avidity of the antibodies, the degree of cross reactivity of the antibodies or the overall concentration of the antibodies in the CSF. Therefore, it is not surprising that there are differing levels of autoantibodies in serum vs CSF of symptomatic subjects, particularly since similar findings have been reported previously in anti-NMDA receptor and other forms of autoimmune encephalitis (64–66).

The results of this investigation suggest that autoantibodies may play a role in the disease pathogenesis of both SC and PANDAS by promoting inflammation and pathological neuronal signaling after crossing the BBB (40, 67). The activation of CaMKII is an important part of normal neuronal signaling cascades, leading to transcriptional activation and synthesis of neurotransmitters such as dopamine (68–72). In SC and PANDAS, antibody-mediated signaling through CaMKII activates tyrosine hydroxylase in neuronal cells and leads to excess dopamine synthesis (9, 73). We can speculate that binding of high avidity, cross-reactive, anti-neuronal autoantibodies to lysoganglioside G_M1_ and dopamine receptors on neurons may lead to pathological alterations in dopamine synthesis and lead to accelerated neurotransmission. Activation of the dopamine receptors or excess synthesis of dopamine may lead to continuous activation of CaMKII or regulate other genes related to disease, which could result in the accumulation of excess extracellular dopamine (73, 74). Studies in our laboratory demonstrate increased dopamine release *in vitro* in the tritiated thymidine dopamine release assay (9). Further, we have shown *in vivo* that intrathecal administration of SC mAb 24.3.1 led to increased tyrosine hydroxylase in neurons in rat brain tissue (9, 13, 75), and expression of SC mAb V gene in transgenic mice demonstrated that the anti-neuronal autoantibodies targeted dopaminergic neurons in the basal ganglia (26). Given the established role of dopamine in movement disorders, including SC and PANDAS, it is not surprising that most acute PANDAS sera (74.3%) were positive for dopamine receptor autoantibodies (D1R and/or D2R). Interestingly, 71.4% of acute PANDAS sera demonstrated positive autoantibodies against D1R and 25.7% against D2R. These results contrast with those from SC wherein six of seven subjects had positive anti-D2R autoantibodies and only two (28.6%) had positive antibodies against D1R (**Figure 1**). Ben-Pazi and colleagues found a similar distribution in a group of SC patients (29). It is tempting to speculate that the differing prevalence of anti-D1R and anti-D2R antibodies in SC and PANDAS may be related to clinical differences between the two disorders. Although both acute and chronic cases may occur in these diseases and the mechanism in these two types of disease may be different, most appear to have at least one elevated autoantibody as evidence of an inflammatory condition.

A recent editorial delineated the steps required to confirm pathogenicity of the autoantibodies in PANDAS (76). In our studies, we document and establish the presence of elevated anti-neuronal autoantibodies in the clinical conditions, both SC and PANDAS, with choreiform (piano-playing) movements. We have shown for some time not only the presence of elevated autoantibodies in serum but also in CSF (8, 13). We now herein confirm the presence of elevated autoantibodies in both serum and CSF in a new group of PANDAS subjects. Secondly, autoantibodies in SC and PANDAS recognize antigens on the surface of the targeted cell and are more likely to be associated with clinical symptoms than autoantibodies binding to intracellular proteins. In addition, we confirm the presence of IgG in the basal ganglia of animal models (34–37) and in transgenic mice or humans expressing autoantibodies from these conditions (24, 26), and that patients with these disorders respond to plasmapheresis with clinical improvement, suggesting autoantibodies play a role in disease (30). Finally, antibodies from animals developing symptoms similar to these disorders can transfer behaviors in animal models (35, 37).

A critical point is how these antibodies are a useful adjunct to clinical diagnosis and identify a basal ganglia encephalitis. For the five tests represented in the anti-neuronal antibody panel, the tests should be positive in affected individuals regardless of the status of commercially available anti-streptococcal antibodies (ASO and anti-streptococcal DNaseB). Anti-neuronal autoantibody ELISA and CaMKII signaling assays demonstrated better sensitivity (91.4%) for the identification of PANDAS than currently clinically available antibody assays, ASO, anti-DNaseB, and ANA. In cases where GAS antibodies are not detectable and psychiatric symptoms and the anti-neuronal autoantibodies are present, Pediatric Acute Onset Neurologic Syndrome or “PANS” would be considered in the differential diagnosis. Anti-neuronal autoantibody titers and antibody-mediated CaMKII activation were elevated in serum and CSF samples taken from acutely ill children meeting diagnostic criteria for PANDAS. Further, the abnormally elevated concentrations of anti-neuronal autoantibodies decreased to normal (negative) levels at 6 months follow-up when more than 80% of the children were much improved (42).

Of the normal subjects examined in this study, 32% had positive results for at least one of the autoantibody ELISA titers, but none had elevated CaMKII signaling activity (**Tables 3** and **5**), suggesting that the autoantibodies in healthy subjects lack ability to signal human neuronal cells. It is well-established that autoantibodies can be elevated for months to years preceding the development of some reported autoimmune syndromes (77, 78), and it is known that autoantibodies can be found in normal unaffected populations due to infections and/or cross-reactivity of autoantibodies with microbial antigens (8, 13, 25, 79). Positivity in a healthy control sample is likely related to cross-reactivity of microbial and host antigens as our previous work has shown (12, 29). This may explain the high rate of positive autoantibodies found by Hesselmark and colleagues in a study of healthy children and adults (80), as they didn't screen for GAS infections. Although the Swedish study found poorer specificity, the sensitivity in their investigation was comparable to the present study, with 100% of PANDAS children having at least one positive autoantibody (80). Results for individual autoantibody assays in both studies were similar, reemphasizing the need to assimilate the complete panel of four antineuronal autoantibodies and CaMKII activation to confirm a diagnosis of PANDAS.

Dale et al. in 2012 reported that 12/17 children with basal ganglia encephalitis (movement and psychiatric disorders) had elevated serum levels of anti-D2R autoantibodies and 10/30 patients with SC, 4/44 with TS, 0/22 with PANDAS, and 0/67 controls had these antibodies (33). They also reported that no patient groups or controls had detectable anti-D1R autoantibodies, which differed from our report here. There are likely technical reasons for these differences including the increased sensitivity of the ELISA compared to the cell-based, flow cytometry assay used in the Dale *et al*. study. The cell-based assay may have not been sensitive enough or epitopes of the antigen recognized in the ELISA were not exposed in the cell-based assays and therefore fewer samples were positive. In addition, problems with control sera may also have prevented the detection of the antibodies in a cell-based assay. Most importantly, signaling in the CaMKII assay or signaling assays of dopamine receptor expressing transfectants have demonstrated functional activation of dopamine receptors by the autoantibodies. The Dale study did not investigate anti-D2R autoantibody function/signaling. Our study herein extends these findings to demonstrate that anti-D1R and anti-D2R autoantibodies are elevated in the sera and CSF by ELISA, and previous studies have confirmed PANDAS-derived autoantibody signaling of D2R in D2R transfectants (26). Current studies suggest that anti-D1R autoantibodies in PANDAS signal a D1R expressing reporter cell line (Menendez and Cunningham, manuscript in preparation). Signaling of D1R and D2R receptors by PANDAS IgG and human mAbs derived from PANDAS supports the hypothesis that functional antibodies which bind and enter human neuronal cells may be important in the pathogenesis of disease. The dopamine receptor antigens used in this study are membrane fragments of the D1 and D2 dopamine receptors, chosen for their maintenance of the receptors' physiological conformation. Previous studies of SC-derived human mAb 24.3.1 bound dopamine receptors as well as sera from SC suggest similar specificity of the human mAb vs sera from the SC patient donor (13, 26, 29). The ratio of anti-dopamine receptor antibody titers correlated with symptoms has already been reported (29). D1R and D2R autoantibody titers were associated with disease outcomes as titers decreased during improvement and increased during worsening symptoms.

Our study suggests that also anti-lysoganglioside antibodies are relevant to disease outcomes. Lysogangioside-G_M1_ is a small molecule shown previously to mimic GLcNAc, the dominant epitope of the group A carbohydrate of *Streptococcus pyogenes* (9). The tubulin protein antigen is purified, which may disrupt the physiological conformation of the protein. A previous study identified specific cross-reactive epitopes of the tubulin protein with GLcNAc (25), the dominant epitope of the group A carbohydrate antigen. However, tubulin does not always track well with episodic changes (**Figures 3C–E**) in PANDAS compared to the other three autoantibodies. Our data suggest that the dopamine receptors as well as lysoganglioside are essential targets in disease and individual symptom presentations depending on the autoantibodies' specificity, cross-reactivity, and avidity.

A limitation of this study is the small number of patients used in all groups. A larger cohort of healthy controls is needed to determine the frequency of positive autoantibodies among children who have had recent GAS infections; such a study was recently completed and data are currently being analyzed (Ben-Pazi, *et al*. manuscript in preparation). To further assess the specificity of the autoantibodies, samples should be obtained from individuals with a wider variety of neuropsychiatric disorders, such as pediatric bipolar disorder, anorexia nervosa, autism, and others. In addition, the sensitivity of the assays in chronic and recurrent PANDAS requires further exploration to determine if autoantibodies remain present throughout the course of illness. If so, the assay will be an important adjunct to the clinical diagnosis of chronic PANDAS, as it is often difficult in the months following onset to determine if neuroinflammation is still playing a role in disease presentation. Anti-neuronal autoantibody profiles and biological mechanisms may be different in the acute and chronic conditions and additional studies are needed to compare them.

With the increased awareness of neuroinflammatory disorders in children, biomarkers are needed that can identify autoantibody-mediated symptom presentations. Our results suggest that the panel of four antineuronal antibodies and CaMKII activation assays successfully identify acute illness in PANDAS, providing opportunities for rapid and accurate diagnosis and treatment. At least one of the panel of autoantibodies was present in elevated concentrations during acute illness in both PANDAS and SC (8, 13, 25, 26), and decreased to normal levels during recovery. Elevation of CaMKII activity suggested that the autoantibodies have bioactivity, consistent with findings of autoimmunity and neuroinflammation in PANDAS and SC (27), including basal ganglia and/or dopamine receptor encephalitis (26, 33). Additional studies are in progress to further investigate the validity of our conclusions, the presence of other neuronal autoantibodies (38), and better understand the pathogenetic mechanisms in disease.

## Data Availability Statement

The datasets generated for this study are available on request to the corresponding author.

## Ethics Statement

University of Oklahoma Health Sciences Center Internal Review Board and the NIMH Internal Review Board reviewed and approved all protocols for the study of human subjects analyzed in this manuscript.

## Author Contributions

JC contributed by supervising methodology, data analysis and interpretation, and writing and revision of the manuscript. KA and AM-B contributed by supervising methodology, performing experiments, and analyzing data. SR and RB contributed by performing experiments. RH and PG contributed by patient recruitment and collection and analysis of data. JL and KW contributed by supervising study design and patient recruitment, collection of data, and revision of the manuscript. IK provided key resources for the completion of the study and revision of the manuscript. JS contributed statistical analyses of the data and wrote statistical sections and provided revision of the manuscript. SS contributed study design, patient recruitment, gathering and interpreting data, acquiring funding, and providing key resources. MC contributed by designing the methodology, supervising and analyzing experiments, acquiring funding, providing resources, and all stages of publication writing and revision of the manuscript. All authors contributed to the article and approved the submitted version.

## Funding

This work was supported (in part) by the Intramural Program of the National Institute of Mental Health of the National Institutes of Health (ZIAMH002666, protocol NTC01281969) and a supplement provided by NHLBI to MC (R01 HL56267) and Mara Family Office, Alberta, Canada.

## Conflict of Interest

MC discloses her affiliation as chief scientific officer/consultant with Moleculera Labs in Oklahoma City where the company offers diagnostic testing for these anti-neuronal autoantibodies in autoimmune neurologic and psychiatric disorders. RB discloses her affiliation with Moleculera Labs as laboratory supervisor and technical lead. She works part time in MC's laboratory at the University of Oklahoma Health Sciences Center where the research laboratory is completely separated physically and financially from Moleculera Labs. RB performed testing on CSF samples from the 35 PANDAS cases in the second cohort. Although SS is a co-inventor on the anti-neuronal autoantibodies/CaMKII panel, neither she nor the NIMH receive any royalties from the patent.

The remaining authors declare that the research was conducted in the absence of any commercial or financial relationships that could be construed as a potential conflict of interest.

## Abbreviations

CSF – Cerebrospinal fluid; PANDAS – Pediatric Autoimmune Neuropsychiatric Disorder Associated with Streptococcal infections; SC – Sydenham chorea; OCD – Obsessive-compulsive disorder; TS – Tourette's syndrome; CaMKII – Calcium/calmodulin-dependent protein kinase II; GAS – Group A beta-hemolytic *Streptococcus*; ADHD – Attention deficit hyperactivity disorder; NIMH – National Institute of Mental Health; IVIG – Intravenous immunoglobulin
